# Unveiling the Younger Face of Gastric Cancer: A Comprehensive Review of Epidemiology, Risk Factors, and Prevention Strategies

**DOI:** 10.7759/cureus.62826

**Published:** 2024-06-21

**Authors:** Kai Kang, Mary Angelica Bagaoisan, YuXin Zhang

**Affiliations:** 1 Institute of Nursing, Angeles University Foundation, Angeles City, PHL; 2 Institute of Clinical Nursing, Gansu Health Vocational College, Lanzhou, CHN

**Keywords:** kap, gastric cancer, younger trend, epidemiology, risk factors, prevention strategies, screening program

## Abstract

Gastric cancer poses a significant global health challenge, with high incidence and mortality rates each year. Despite advancements in screening and treatment, late detection remains a critical issue. Efforts to address this include raising public awareness and implementing targeted screening programs for high-risk populations. The increasing incidence of gastric cancer among younger individuals underscores the need for lifestyle adjustments and targeted interventions to mitigate risks and improve outcomes. Understanding the various factors contributing to gastric cancer risk is essential for effective prevention strategies, including Helicobacter pylori eradication, lifestyle modifications, and regular screening for high-risk groups. A comprehensive approach addressing both individual behaviors and broader societal factors is crucial in the fight against gastric cancer.

This review provides an in-depth examination of gastric cancer epidemiology, risk factors, preventive measures, and screening initiatives, with a particular focus on the rising incidence among younger demographics. Emphasizing the importance of early detection and intervention, the review highlights the need for proactive screening to improve patient outcomes and reduce mortality rates. By addressing these aspects comprehensively, this paper aims to enhance the understanding of gastric cancer dynamics, particularly its incidence among younger individuals, and to inform future strategies for prevention and control.

## Introduction and background

Gastric cancer, a prevalent malignant tumor globally, poses a significant health challenge. The International Agency for Research on Cancer reported approximately 1.089 million new cases and 769,000 deaths from gastric cancer in 2020, ranking it fifth in global cancer incidence and fourth in mortality [1]. By 2030, gastric cancer is expected to become a leading cause of death worldwide, with an estimated 2.5 million new cases and 1.9 million deaths by 2050 [2]. In China, gastric cancer is a major health burden, ranking third in both incidence and mortality among all cancers [3]. The country accounted for 479,000 new cases and 374,000 deaths in 2020 alone [4].

Gastric cancer primarily affects individuals over 50 years old and shows a male predominance, with a male-to-female ratio of approximately 2:1 [5]. Despite a global decline in overall incidence and mortality rates due to advancements in diagnosis and treatment, there has been a concerning rise in gastric cancer incidence among younger populations, with 4.4% to 9.8% of cases occurring in individuals under 40 years old [6]. Young patients often experience delayed diagnosis due to less conspicuous symptoms, leading to advanced disease stages and poorer prognoses [7]. These trends underscore the urgent need for targeted intervention strategies.

Recent studies have highlighted distinctive clinicopathological features in young gastric cancer patients, including a higher proportion of females, poorly differentiated tumors, and a propensity for locally advanced or advanced-stage disease [8]. While traditional risk factors such as *Helicobacter pylori *(HP) infection, dietary habits, and genetic predisposition remain relevant, a broader examination encompassing symptoms, preventive measures, and management strategies is warranted [9].

This literature review provides a comprehensive analysis of the epidemiology, risk factors, prevention strategies, and future research directions concerning gastric cancer, with a particular focus on the rising incidence among younger populations. Understanding these facets is crucial for devising effective preventive measures and improving clinical outcomes in gastric cancer management.

## Review

Gastric cancer: epidemiology

The World Health Organization's (WHO) latest report in 2020 underscores the global burden of gastric cancer, with over 1 million cases diagnosed and more than 768,000 deaths. Gastric cancer is now the fifth most common cancer globally and the third leading cause of cancer-related deaths [10]. However, the incidence of gastric cancer shows significant regional disparities, with Northeast Asia, South America, Central America, and Eastern Europe bearing the highest disease burden [11,12]. Particularly alarming are the incidence rates in Japan and South Korea, especially among men, while China grapples with high mortality rates, positioning gastric cancer as one of the deadliest cancers in the country [11-13]. Conversely, Western Europe, sub-Saharan Africa, Australia, and North America report relatively lower incidence rates [12].

Over the past few decades, gastric cancer rates have declined significantly in North America and Western Europe, along with increased access to clean drinking water, improved food preservation, better dietary habits, and eradication of HP [14]. However, this decline is primarily seen in the distal gastrointestinal type of gastric cancer. The incidence of proximal diffuse gastric cancer is still increasing in North America and Western Europe, for reasons that are currently unknown [14,15]. Except in high-income countries, distal gastric cancer still dominates the proportion of gastric cancer worldwide [16]. Gastric cancer has a poor prognosis because it is usually at an advanced stage when detected. Currently, the most effective prevention method is early screening, which involves early detection, diagnosis, and treatment to improve the prognosis of gastric cancer patients and reduce mortality [15,17].

Narii et al. [17] conducted a large prospective population-based cohort study including 80,272 participants. They demonstrated that early screening reduced the incidence of advanced gastric cancer and gastric cancer mortality. The results showed that in the endoscopic screening group, gastric cancer mortality and advanced gastric cancer incidence were reduced by 61% (hazard ratio (HR) = 0.39 (95% CI: 0.30-0.51)) and 22% (HR = 0.78 (95% CI: 0.67-0.90)), respectively. X-ray screening reduced gastric cancer mortality (HR = 0.63 (95% CI: 0.54-0.73)), but its effectiveness was lower than that of endoscopic screening.

According to the latest data from China in 2020, the incidence and mortality rates of gastric cancer remain high, ranking third among all malignant tumors [3]. In China, there are approximately 480,000 new cases of gastric cancer each year, with only about 20% of these being early gastric cancer cases. Most patients are diagnosed at an advanced stage, resulting in an overall five-year survival rate of less than 50% [18]. The regions with high gastric cancer incidence are widely distributed, with concentrations in northwest China and southeast coastal areas. These regions also include scattered high-incidence areas, with rural areas exhibiting higher rates than urban areas. Data show that the Liaodong Peninsula, Shandong Peninsula, Yangtze River Delta, Taihang Mountains, and other areas are high-incidence regions for gastric cancer [19,20]. The provinces with the highest incidence of gastric cancer include Liaoning, Fujian, Gansu, Shandong, and Jiangsu [21]. With the increased use of endoscopy, the proportion of early gastric cancer diagnoses is rising year by year [22].

Currently, the main methods and strategies for treating gastric cancer involve comprehensive treatments based on surgery. This includes the planned and reasonable application of surgery, chemotherapy, radiotherapy, biological targeting, and other therapeutic approaches to radically cure or control tumors, prolong patients' survival periods, and improve their quality of life [18].

An effective way to prevent gastric cancer in China is through extensive early screening. Gastric cancer screening can significantly improve the detection rate of early gastric cancer lesions, enhance patient prognosis, and greatly improve survival rates [18,22]. However, the low level of public awareness of gastric cancer and its screening restricts the effectiveness and benefits of early detection, diagnosis, and treatment [22]. Efforts to increase public education and awareness are imperative to optimize the impact of gastric cancer screening programs in China.

Gastric cancer: younger trend

Gastric cancer, a prevalent malignancy of the digestive system, predominantly affects middle-aged and elderly individuals. However, a concerning trend has emerged with the increasing incidence of gastric cancer among young people in recent years, indicating a shift towards a younger demographic. Currently, there exists no universally accepted standard for classifying gastric cancer in young individuals. Some studies define young-onset gastric cancer as cases diagnosed before the age of 40 [23,24]. Although the highest proportion of gastric cancer patients worldwide is in the 50-70 age group, the proportion of younger people is small but still considerable, accounting for approximately 2.0%-6.2% of the total incidence of gastric cancer [25,26]. Surprisingly, while the overall incidence of gastric cancer worldwide has been declining, the incidence among young individuals has either stabilized or shown an upward trajectory [27-29]. Islami et al. [28], using high-quality United States nationwide population-based data to calculate the average contemporary incidence (2010-2014) and percentage change in annual incidence (1997-2014) for major subtypes of gastric cancer by race, ethnicity, and age, found increasing incidence trends for gastric cardia adenocarcinoma and gastric non-cardia adenocarcinoma in several subpopulations in the United States. Yin et al. [27], exploiting a National Disease Surveillance System published by the China Center for Disease Control and Prevention (CDC) to investigate the epidemiological features of gastric cancer-related mortality in China between 2006 and 2013, found that crude and standardized rates of the 0-29 years old group increased by 22.3% and 16.2%, respectively. The results reveal a remarkable increase in gastric cancer-related mortality in subjects under the age of 30, calling for further measures to prevent the increase in the incidence of gastric cancer in young patients.

Young-onset gastric cancer presents distinctive biological characteristics and clinicopathological features that warrant attention. These include a higher prevalence among females, poorly differentiated tumors, and a propensity towards locally advanced or advanced disease stages [8]. Notably, young female patients exhibit a higher positive rate of estrogen receptors, implicating estrogen exposure as a significant contributor to gastric cancer development [30]. Tumors in young individuals tend to manifest in the fundus or antrum of the stomach, predominantly as diffuse-type gastric cancer with poor or undifferentiated differentiation.

Signet ring cell carcinoma, characterized by a higher degree of malignancy, is particularly prevalent among young gastric cancer patients. This phenomenon may be linked to dysregulated expression of E-cadherin resulting from CDH1 mutation, contributing to increased invasiveness, metastatic potential, and disease progression [31,32]. Factors such as HP infection, genetic variations, nonspecific clinical symptoms, and delayed diagnosis may further exacerbate disease progression in young adults [33,34]. Locally advanced disease stages and lymph node or distant metastases are more frequently observed in young patients, with some individuals deemed ineligible for radical resection at the time of diagnosis, potentially contributing to poorer prognosis compared to their middle-aged and elderly counterparts [35]. However, this perspective remains subject to debate. Niu et al. [8] highlighted that the prognosis of young patients with pTNMH stage I gastric cancer may be worse than that of middle-aged and elderly patients, while no age-specific differences were observed in other disease stages.

Gastric cancer: risk factors

The occurrence of gastric cancer is a complex process with multifactor participation and multistep evolution, which is the comprehensive result of the interaction between genetic and environmental factors.

HP Infection

Since 1994, the WHO's International Agency for Research on Cancer (IARC) has recognized HP as a class I carcinogen. Its prolonged presence in the stomach is known to induce chronic gastritis and significantly contribute to the development of gastric mucosal atrophy and intestinal metaplasia [36]. In 2019, the Chinese Expert Consensus on *Helicobacter pylori* Eradication and Prevention and Control of Gastric Cancer highlighted HP infection as the primary cause of gastric cancer in China, emphasizing that HP eradication can effectively reduce the risk of gastric cancer in the country [37].

A meta-analysis by Eslick et al. [38], comprising 42 studies, demonstrated a notable association between HP infection and gastric cancer risk. The results revealed that HP-infected individuals faced a 2.04-fold higher risk of developing gastric cancer compared to non-infected counterparts (odds ratio (OR) = 2.04, 95% confidence interval (CI): 1.69-2.45). Importantly, HP infection appears to be intricately linked to gastric cancer onset in young individuals, who predominantly present with poorly differentiated or undifferentiated tumors [30,39]. HP infection fosters a pro-inflammatory environment and promotes intestinal metaplasia, thereby facilitating the emergence of undifferentiated gastric cancer in this demographic.

Familial clustering of HP infection is a notable phenomenon, with high rates observed among parents and siblings of young individuals diagnosed with gastric cancer. This familial aggregation suggests a potential mode of transmission from parents to children during infancy or between siblings, underscoring the intimate connection between familial HP infection and the development of gastric cancer in young individuals [40]. These findings emphasize the critical role of HP eradication strategies in not only reducing the overall burden of gastric cancer but also in mitigating its occurrence among younger populations with a predisposition to familial HP infection.

Diet and Eating Habits

Dietary choices and eating habits play a pivotal role in the development of gastric cancer, constituting a significant aspect of its pathogenesis. These habits encompass a spectrum of factors including excessive salt consumption, regular intake of cured, smoked, grilled, and fried foods, frequent consumption of red and processed meats, inadequate consumption of fresh fruits and vegetables, and poor dietary routines [41].

According to the World Cancer Research Fund (WCRF), excessive salt intake stands out as a prominent risk factor for gastric cancer. While the human body requires salt in moderation, overconsumption can stimulate gastric mucosa, potentially leading to mucosal atrophy, increased DNA synthesis, and cellular proliferation, thus facilitating the onset of gastric cancer [42]. A meta-analysis by D’Elia et al. synthesized findings from seven studies, demonstrating a 1.68 times higher risk of gastric cancer among individuals with high salt intake compared to those with lower intake levels (RR=1.68, 95%CI: 1.17-2.41) [43].

The long-term consumption of pickled, smoked, fried, grilled, and cured foods, such as salted fish, pickles, and barbecued items, can elevate the risk of gastric cancer. During the production of such foods, carcinogens like polycyclic aromatic hydrocarbons (PAHs) and N-nitroso compounds are formed, which have been consistently associated with gastric cancer in various studies [44,42]. Cheng et al. conducted a comprehensive meta-analysis encompassing 39 studies, six of which focused on fried foods, revealing a significant association between fried food consumption and gastric cancer risk in the Chinese population (OR=2.28, 95%CI: 1.87-2.78) [45].

Consumption of red meat and processed meat is also linked to an elevated risk of gastric cancer [46,47]. A meta-analysis by Kim et al. [48] consolidated data from 43 studies, including 11 cohort studies and 32 case-control studies, indicating that both red meat (RR=1.41, 95%CI: 1.21-1.66) and processed meat (RR=1.57, 95%CI: 1.37-1.81) consumption correlate with increased gastric cancer risk. Moreover, for every 100g/d increment in red meat consumption, the risk of gastric cancer rose by 26% (RR=1.26, 95%CI: 1.11-1.42), and for every 50g/d increase in processed meat consumption, the risk surged by 72% (RR=1.72, 95%CI: 1.36-2.18).

Evidence suggests that a higher intake of vegetables and fruits can mitigate the risk of gastric cancer [49,46,42]. Zhang et al.'s meta-analysis revealed that compared to individuals with low dietary fiber intake, those with high dietary fiber intake experienced a 42% risk reduction (OR=0.58, 95%CI: 0.49-0.67) for gastric cancer, with a further 44% risk reduction (OR=0.56, 95%CI: 0.46-0.67) for every 10g/d increment in dietary fiber intake [50]. Moreover, studies indicate that consumption of citrus fruits [51] and vegetables rich in vitamin C, as well as onions and garlic, can lower the risk of gastric cancer [52].

Unhealthy eating habits can induce recurrent damage and repair cycles in gastric mucosa, compromising its protective mechanisms and potentially leading to carcinogenesis. Zhou et al.'s meta-analysis among the Chinese population identified several eating habits as risk factors for gastric cancer, including skipping breakfast, irregular meal patterns, fast eating, overeating, and consuming leftovers [53]. These habits, when persistent over the long term, can contribute to the development of gastric cancer.

Smoking

Smoking is widely recognized as a significant contributor to the development of gastric cancer, with numerous studies indicating a clear dose-response relationship, wherein the risk of gastric cancer escalates proportionally with both the intensity and duration of smoking [54-56]. This correlation underscores the importance of understanding the profound impact of tobacco use on gastrointestinal health.

In a seminal meta-analysis conducted by Ordóñez-Mena and colleagues [57], data from 19 prospective cohort studies involving a staggering 897,021 participants from Europe and the United States were meticulously analyzed. Through rigorous adjustments for potential confounders such as age, gender, and body mass index (BMI), the study revealed alarming statistics. Current smokers were found to face a striking 1.74-fold increase in the risk of developing gastric cancer (HR=1.74, 95%CI: 1.50-2.02) compared to non-smokers. Even among former smokers, who had ceased the habit, there remained a noteworthy elevation in risk, with a 1.18-fold increase observed (HR=1.18, 95%CI: 0.95-1.46). These findings underscore the enduring impact of tobacco exposure on gastric health, persisting even after smoking cessation.

Building upon this foundation of evidence, Poorolajal et al. [58] conducted an extensive meta-analysis encompassing a vast array of studies spanning over three decades, from 1985 to 2018. Their comprehensive analysis corroborated and extended the findings of previous research, reaffirming the deleterious association between smoking and gastric cancer risk. Current smokers were found to be at a substantial 1.61-fold increased risk compared to their non-smoking counterparts (OR=1.61, 95%CI: 1.49-1.75), underscoring the profound impact of tobacco use on gastric carcinogenesis. Furthermore, even among former smokers, the risk remained significantly elevated, with a 1.43-fold increase observed (OR=1.43, 95%CI: 1.29-1.59). This persistent risk highlights the enduring legacy of tobacco exposure, necessitating ongoing efforts to raise awareness and implement effective smoking cessation interventions.

These findings carry significant public health implications, emphasizing the urgent need for comprehensive tobacco control measures aimed at curbing smoking prevalence and mitigating its associated health burdens. Strategies such as increased taxation, public education campaigns, and access to smoking cessation programs are essential in addressing this pressing public health challenge. By addressing tobacco use comprehensively, we can mitigate the substantial burden of gastric cancer and improve the overall health and well-being of populations worldwide.

Alcohol Consumption

Alcohol consumption stands as a recognized risk factor in the complex landscape of gastric cancer etiology. The impact of alcohol on gastric health is multifaceted, with factors such as the type of alcohol consumed, the quantity ingested, and the duration of consumption contributing to its effects, albeit with inconclusive consensus in existing literature. Nonetheless, studies consistently highlight the heightened risk of gastric cancer associated with heavy alcohol consumption [59-61].

In a seminal meta-analysis by Tramacere et al. [62], data synthesized from 59 studies, incorporating a substantial cohort of 34,557 gastric cancer cases, provided valuable insights. Their findings revealed that individuals who consumed alcohol faced a modest yet discernible 1.07-fold increase in the risk of gastric cancer compared to non-drinkers (RR=1.07, 95%CI: 1.01-1.13). Furthermore, the risk escalated significantly among heavy drinkers, defined as those consuming ≥50g of alcohol per day, with a 1.20-fold increase observed (RR=1.20, 95%CI: 1.01-1.44) compared to non-drinkers. These results underscore the dose-dependent relationship between alcohol consumption and gastric cancer risk, with heavier consumption magnifying the associated health hazards.

Building upon this evidence base, Rota et al. [63] conducted a comprehensive meta-analysis focusing on 20 European studies, encompassing a sizable cohort of 9,669 cases and 25,336 controls. Their analysis provided further clarity on the nuanced association between alcohol intake and gastric cancer risk. Comparing drinkers to non-drinkers/ex-drinkers, the study revealed a progressive increase in risk with escalating alcohol consumption. Heavy drinkers, defined as those consuming 48-72 g of alcohol per day, exhibited a notable 26% increase in gastric cancer risk (OR=1.26, 95%CI: 1.08-1.48), while extremely heavy drinkers, consuming over 72g of alcohol per day, faced an even more substantial 48% increase in risk (OR=1.48, 95%CI: 1.29-1.70). These findings elucidate the dose-response relationship between alcohol consumption and gastric cancer, with heavier intake correlating with a heightened risk profile.

The implications of these findings are profound, underscoring the importance of addressing alcohol consumption patterns as part of comprehensive strategies to mitigate gastric cancer risk. Public health initiatives aimed at raising awareness about the detrimental effects of excessive alcohol consumption, coupled with policies promoting moderation and access to support services for individuals struggling with alcohol dependency, are crucial in stemming the tide of alcohol-related gastric malignancies. By addressing alcohol consumption within the broader context of preventive health measures, we can strive towards reducing the burden of gastric cancer and improving overall population health outcomes.

Family History of Gastric Cancer

The familial aggregation of gastric cancer, particularly within first-degree relatives (FDR), stands as a well-documented risk factor in the intricate landscape of gastric malignancy [46,64]. Jiang et al. [65] shed light on this association, demonstrating that distal gastric adenocarcinoma exhibits a notable correlation with a family history of gastric cancer, with an odds ratio of 2.15 (95% CI: 1.18-3.91). This finding underscores the genetic predisposition inherent in certain familial contexts, implicating inherited factors in the pathogenesis of gastric adenocarcinoma.

Delving deeper into the familial dynamics of gastric cancer, a retrospective cohort study conducted by Reddy et al. [66] in the United States yielded compelling insights. Their investigation among patients with intestinal metaplasia unveiled a stark reality: a family history of gastric cancer conferred a substantial 3.80-fold increase in the risk of developing gastric malignancies (HR=3.80, 95% CI: 1.50-9.70). This robust association highlights the profound impact of familial predisposition on individual susceptibility to gastric cancer, emphasizing the importance of familial history assessment in risk stratification and preventive interventions.

Furthermore, emerging evidence suggests that the risk of gastric cancer is significantly heightened in families with a history of gastric cancer or other digestive system malignancies, particularly among young and middle-aged individuals [67]. Common genetic predisposing syndromes implicated in this familial clustering include hereditary nonpolyposis colon cancer, familial adenomatous polyposis, and Peutz-Jeghers syndrome, among others. These syndromes underscore the intricate interplay between genetic susceptibility and environmental factors in gastric carcinogenesis, offering valuable insights into the multifactorial nature of familial gastric cancer predisposition.

The implications of these findings extend beyond individual risk assessment, shaping clinical management strategies and public health interventions aimed at early detection and prevention. Incorporating family history assessment into risk stratification protocols enables targeted surveillance and screening efforts among high-risk populations, facilitating early detection and intervention. Moreover, fostering genetic counseling services and promoting awareness of familial risk factors empowers individuals and families to make informed decisions regarding their healthcare and lifestyle choices. By elucidating the complex interplay between genetic predisposition and environmental influences, we can advance toward personalized approaches to gastric cancer prevention and management, ultimately reducing the burden of this devastating disease on individuals and communities alike.

Gastric cancer prevention

Gastric cancer, a prevalent malignant tumor originating from the epithelial lining of the gastric mucosa, poses a significant public health challenge worldwide. Afflicting predominantly individuals aged 50 and above, its incidence rates are alarmingly high, reflecting a pressing health concern [68]. Moreover, shifting dietary patterns, escalating work-life stressors, the pervasive influence of Helicobacter pylori (HP) infection, genetic predispositions, and environmental factors collectively contribute to a notable surge in gastric cancer prevalence. Notably, the disease's epidemiological landscape is evolving, with a concerning trend towards younger onset, marked by high mortality rates and poor prognoses.

Efforts to combat the escalating burden of gastric cancer underscore the pivotal role of early intervention in residents' lifestyles and the dissemination of pertinent disease-related knowledge [69]. Empowering individuals with heightened awareness regarding gastric cancer risks and preventive measures holds promise in augmenting clinical management strategies and averting disease onset. Of particular significance is the eradication of HP infection, a key preventive measure that not only curtails gastric cancer incidence but also mitigates the risk of associated conditions such as chronic atrophic gastritis and peptic ulcer disease [70]. Wong et al. [71] advocate for targeted interventions focusing on HP infection prevention, positing its efficacy in reducing gastric cancer incidence, particularly among young adults.

Moreover, lifestyle modifications, including dietary adjustments, emerge as cornerstone strategies in gastric cancer prevention. Avoidance of high-salt diets and reduction in the consumption of pickled, smoked, fried, grilled, or processed foods are advocated as prudent measures [42,44]. Likewise, curbing red and processed meat intake, while augmenting the consumption of fresh vegetables and fruits, embodies a sensible dietary approach endorsed by multiple studies [46,47,49]. Adopting regular eating patterns, coupled with smoking cessation and abstinence from alcohol, further diminishes gastric cancer risk [53-56,59-61].

For individuals harboring a family history of gastric cancer, proactive screening initiatives assume paramount importance. Routine gastric cancer screenings facilitate early detection, diagnosis, and timely interventions, thereby enhancing treatment outcomes and prognosis [14]. By leveraging comprehensive screening protocols, healthcare providers can identify high-risk individuals and implement tailored surveillance strategies, thereby mitigating the impact of familial predispositions on disease progression.

In conclusion, a multifaceted approach encompassing lifestyle modifications, HP infection eradication, and proactive screening initiatives is imperative in combating the escalating burden of gastric cancer. Through concerted efforts aimed at raising awareness, promoting preventive measures, and fostering early detection strategies, we can collectively strive towards curbing the incidence and mortality associated with this formidable malignancy, ultimately enhancing public health outcomes on a global scale.

Gastric cancer programs in China

Since 2013, China has vigorously promoted the construction of a regional medical alliance model, with tertiary hospitals as the core, and other medical institutions in a certain region, including primary health service centers, integrating public health resources in the region, and giving full play to the leading role of core hospitals. A hierarchical medical model of "minor diseases in the community, serious diseases in the hospital, and rehabilitation back to the community" should be established [72]. However, as the construction of the regional medical alliance model is in full swing, its promotion has also encountered many bottlenecks and difficulties. The next step of the regional medical alliance model has become the focus of more and more professionals [73,74]. In addition, China is currently facing health problems caused by industrialization, urbanization, an aging population, disease spectrum changes, and evolving lifestyles. With the promulgation of "Healthy China 2030" and "China's Long-term Plan for the Prevention and Control of Non-communicable Diseases (2017-2025)," both initiatives emphasize the development of the health service industry and the transition from a "treatment-centered" to a "health-centered" approach, opening a new era in the construction of a Healthy China.

In this situation, the health management consortium came into being. The health management alliance is a health-oriented health management service system with the goal of "prevention of serious diseases, management of chronic diseases, and promotion of health," with medical and health management service institutions as the main body [72]. The health management alliance can integrate the resources of health management institutions in the region, serving as an important complementary form of the regional medical alliance model and playing a crucial role in the improvement and development of the medical and health service system.

The Gastric Cancer Prevention and Health Management Consortium is a serious disease prevention and screening alliance for gastric cancer conducted in China's National Health Management Demonstration Base, with the purpose of screening relevant high-risk groups and carrying out early intervention. It is necessary and meaningful to take advantage of the screening capabilities of health management centers at all levels to carry out the prevention and control of gastric cancer in healthy people. The goal of building the gastric cancer prevention and health management consortium is to leverage the screening advantages of health management centers at all levels to conduct gastric cancer prevention and control in healthy individuals. By advancing the examination threshold, patients can receive timely examinations and diagnoses in the early stages of gastric cancer, enabling early treatment, improving survival rates and quality of life, and reducing the incidence of cancer in China while enhancing the cure rate.

In China, the battle against gastric cancer hinges on targeted interventions aimed at high-risk populations, defined as individuals with an elevated susceptibility to developing the disease. The identification of these high-risk groups represents a pivotal step in disease prevention and control, facilitating early detection, diagnosis, and timely treatment interventions. However, the absence of simple and efficient diagnostic modalities for early gastric cancer screening poses a significant challenge within the Chinese healthcare landscape. Factors such as limited healthcare resources, financial constraints, and low acceptance among asymptomatic individuals and those not deemed high-risk impede the widespread implementation of methods such as gastroscopy. Consequently, a pragmatic approach prioritizing gastric cancer screening for high-risk populations emerges as a viable strategy [22].

The "Consensus on Early Gastric Cancer Screening and Endoscopic Diagnosis and Treatment in China (April 2014, Changsha)" authored by Liao et al. [75] delineates the criteria defining individuals at high risk of gastric cancer. These criteria include individuals aged 40 or above meeting any of the following: residence in high-risk areas, HP infection, presence of chronic atrophic gastritis, gastric ulcer, gastric polyps, or a history of gastric cancer in first-degree relatives, among other pre-gastric diseases. Additionally, individuals exposed to other risk factors such as high-salt diets, pickled foods, smoking, and heavy alcohol consumption fall within this high-risk category.

Furthermore, HP detection emerges as a cornerstone of gastric cancer screening protocols, as elucidated in the "Expert Consensus on Screening Procedures for Early Gastric Cancer in China (Draft) (2017, Shanghai)" by Du et al. [76]. Serological HP detection, performed concurrently with pepsinogen (PG) and gastrin (G-17) detection, offers a non-invasive and feasible screening method, circumventing the compliance issues associated with alternate HP detection methods such as stool collection or gastric mucosal biopsy. The urea breath test (UBT) serves as a supplementary tool for HP antibody-positive patients, offering additional diagnostic clarity in regions where resources permit. Moreover, the detection of gastric cancer biomarkers, including PG, G17, MG7-Ag, and serum HP-Ag, in conjunction with a risk stratification scoring system, facilitates the identification and concentration of high-risk populations suitable for targeted examination. However, the economic viability of these approaches warrants careful consideration [22].

The prevailing gastric cancer screening strategy in China involves the initial utilization of non-invasive diagnostic methods to identify high-risk cohorts, followed by purposeful endoscopic evaluation [76]. According to the risk classification of gastric cancer, patients with grade A, pepsinogen PG (-), and HP (-) do not need endoscopy. Patients with grade B, PG (-), and HP (+) should undergo endoscopy at least every three years. Endoscopy should be performed at least once every two years in patients with grade C, PG (+), and HP (+). Patients with grade D, PG (+), and HP (-) should undergo endoscopy once a year [75].

In essence, the strategic deployment of screening resources tailored to high-risk populations holds promise in bolstering early detection efforts and optimizing clinical outcomes in the fight against gastric cancer in China. Through a judicious combination of non-invasive screening modalities and targeted endoscopic surveillance, healthcare providers can effectively prioritize resources and maximize the impact of gastric cancer prevention and control initiatives.

Future research perspectives

Gastric cancer stands as a formidable adversary, characterized by a protracted and intricate pathogenesis influenced by a myriad of factors and unfolding through multi-step evolution. Clinical investigation data underscore the significance of various lifestyle elements such as irregular diet, smoking, family history of gastric cancer, high-salt diet, sedentary behavior, alcohol consumption, and HP infection in fueling its onset and progression [77,78]. Alarmingly, approximately 59.8% of gastric cancer cases are attributed to lifestyle choices, highlighting the profound impact of modifiable risk factors. The pivotal role of lifestyle modifications in preventing cancer is evident, with timely corrections and the adoption of healthy dietary and lifestyle practices estimated to avert 30%-50% of cancer incidences.

Given the substantial contribution of lifestyle factors to gastric cancer risk, early intervention targeting residents' living environments and behaviors assumes paramount importance in primary prevention efforts. By empowering individuals to make informed choices and cultivate healthier habits, healthcare initiatives can significantly curtail the burden of gastric cancer and other malignancies. Central to this approach is an understanding of residents' perceptions regarding gastric cancer risk factors, as it serves as a crucial determinant of their engagement in cancer screening endeavors [69]. Early identification of these perceptions provides invaluable insights for the development and implementation of targeted prevention and control strategies, fostering a proactive approach to disease management.

Despite overall declines in gastric cancer incidence and mortality rates globally, a concerning trend has emerged with the rising incidence of gastric cancer among young people. These patients, often overlooked due to subtle symptoms, face delayed diagnosis and, consequently, poorer prognoses upon reaching advanced stages [7]. Recognizing the unique challenges posed by gastric cancer in young individuals is imperative, necessitating swift action to enhance their awareness and understanding of the disease. Educational initiatives targeting young populations can play a pivotal role in dispelling misconceptions and promoting healthy lifestyle choices, thereby reducing the incidence of gastric cancer in this demographic.

Moreover, proactive measures to improve self-prevention awareness and encourage regular gastric cancer screenings among high-risk young individuals are imperative. By fostering a culture of preventive healthcare and early detection, healthcare providers can mitigate the impact of gastric cancer in young populations and ensure timely interventions when needed [76]. This approach not only facilitates precision prevention but also enables early screening and effective treatment, laying a solid foundation for primary prevention efforts among adolescents.

In conclusion, addressing the complex interplay of lifestyle factors and enhancing awareness among young individuals are critical components of effective gastric cancer prevention strategies. Through targeted educational initiatives, lifestyle interventions, and proactive screening efforts, healthcare systems can empower individuals to take charge of their health and mitigate the burden of gastric cancer across all age groups [75]. By fostering a culture of prevention and early intervention, we can strive towards a future where gastric cancer incidence is minimized, and outcomes are optimized for all individuals, regardless of age.

## Conclusions

Gastric cancer is a major health challenge facing the world, with a high number of cases and mortality rates diagnosed each year. Despite advancements in screening and treatment, late detection remains a critical issue. Efforts to combat this include raising public awareness and implementing targeted screening programs for high-risk populations. Additionally, the concerning rise in gastric cancer incidence among younger individuals highlights the importance of lifestyle adjustments and targeted interventions to reduce risks and improve outcomes. Understanding the various factors contributing to gastric cancer risk is essential for effective prevention strategies, requiring interventions such as HP eradication, lifestyle modifications, and regular screening for high-risk groups. A comprehensive approach that addresses both individual behaviors and broader societal factors is crucial in the fight against gastric cancer.

Despite improvements in lifestyle and the eradication of HP leading to a decrease in overall gastric cancer incidence in some regions, there is a troubling increase in cases among younger age groups. This review offers a thorough examination of gastric cancer epidemiology, risk factors, preventive measures, and screening initiatives, with a particular focus on this emerging trend among younger demographics. Emphasizing the significance of early detection and intervention, the review underscores the importance of proactive screening to enhance patient outcomes and reduce mortality rates. By addressing these aspects comprehensively, this paper aims to enhance understanding of gastric cancer dynamics, especially its incidence among younger individuals, and inform future strategies for prevention and control.
